# Purines and Carotid Body: New Roles in Pathological Conditions

**DOI:** 10.3389/fphar.2017.00913

**Published:** 2017-12-12

**Authors:** Silvia V. Conde, Emilia C. Monteiro, Joana F. Sacramento

**Affiliations:** Centro de Estudos de Doenças Crónicas, NOVA Medical School – Faculdade de Ciências Médicas, Universidade NOVA de Lisboa, Lisboa, Portugal

**Keywords:** carotid body, adenosine, ATP, hypertension, chronic intermittent hypoxia, type 2 diabetes

## Abstract

It is known that adenosine and adenosine-5′-triphosphate (ATP) are excitatory mediators involved in carotid body (CB) hypoxic signaling. The CBs are peripheral chemoreceptors classically defined by O_2_, CO_2_, and pH sensors. When hypoxia activates the CB, it induces the release of neurotransmitters from chemoreceptor cells leading to an increase in the action potentials frequency at the carotid sinus nerve (CSN). This increase in the firing frequency of the CSN is integrated in the brainstem to induce cardiorespiratory compensatory responses. In the last decade several pathologies, as, hypertension, diabetes, obstructive sleep apnea and heart failure have been associated with CB overactivation. In the first section of the present manuscript we review in a concise manner fundamental aspects of purine metabolism. The second section is devoted to the role of purines on the hypoxic response of the CB, providing the state-of-the art for the presence of adenosine and ATP receptors in the CB; for the role of purines at presynaptic level in CB chemoreceptor cells, as well as, its metabolism and regulation; at postsynaptic level in the CSN activity; and on the ventilatory responses to hypoxia. Recently, we have showed that adenosine is involved in CB hypersensitization during chronic intermittent hypoxia (CIH), which mimics obstructive sleep apnea, since caffeine, a non-selective adenosine receptor antagonist that inhibits A_2A_ and A_2B_ adenosine receptors, decreased CSN chemosensory activity in animals subjected to CIH. Apart from this involvement of adenosine in CB sensitization in sleep apnea, it was recently found that P2X3 ATP receptor in the CB contributes to increased chemoreflex hypersensitivity and hypertension in spontaneously hypertension rats. Therefore the last section of this manuscript is devoted to review the recent findings on the role of purines in CB-mediated pathologies as hypertension, diabetes and sleep apnea emphasizing the potential clinical importance of modulating purines levels and action to treat pathologies associated with CB dysfunction.

## Purines Metabolism

From all purines, adenosine and adenosine-5′-triphosphate (ATP) are the only ones that are known to have a role in cell to cell communication and therefore they act extracellularly to mediate several biological effects via cell-surface receptors, the purine receptors. ATP has a fundamental intracellular role as universal source of energy for all living cells. The demonstration of its release into the extracellular space and the identification and localisation of specific receptors on target cells have been essential in establishing its extracellular physiological role. In the beginning of the seventies, the purinergic neurotransmission was first proposed by Burnstock (1972). ATP was shown to be released from non-adrenergic, non-cholinergic nerves to signaling inhibitory enteric nerves in the guinea pig taenia coli and excitatory parasympathetic nerves in the urinary bladder (Burnstock et al., 1970, 1972). However, the concept of purinergic neurotransmission was only established in the nineties, when receptors for adenosine and ATP were cloned and sequenced (for a review see Ralevic and Burnstock, 1998). Short-term purinergic signaling was first described when ATP was identified as a cotransmitter with noradrenalin, acetylcholine and with substance P and calcitonin gene-related peptide (for a review see Burnstock, 2016) in the peripheral nervous system. Later ATP was shown to be a cotransmitter in neurons in the central nervous system (CNS), being co-released with GABA (Jo and Schlichter, 1999; Jo and Role, 2002) and Glutamate (Pankratov et al., 1999). Adenosine is a product of ATP catabolism, which can be used to resynthesize ATP itself. This mediator is an ubiquitous substance that is not stored or released as a classical neurotransmitter, being released by almost all cell types through nucleoside transporters (Fredholm et al., 2001). Intracellularly it has key roles in pathways as purinergic nucleic acid base synthesis, amino acid metabolism and modulation of cellular metabolic status (Conde et al., 2009). Extracellularly, adenosine modulates the activity of several systems at presynaptic level (inhibiting or facilitating neurotransmitters release), at postsynaptic or at non-synaptic level (e.g., modulating blood flow or the metabolism of sustentacular cells).

### Metabolic Pathways of Adenosine Formation and Release

**FIGURE 1:**
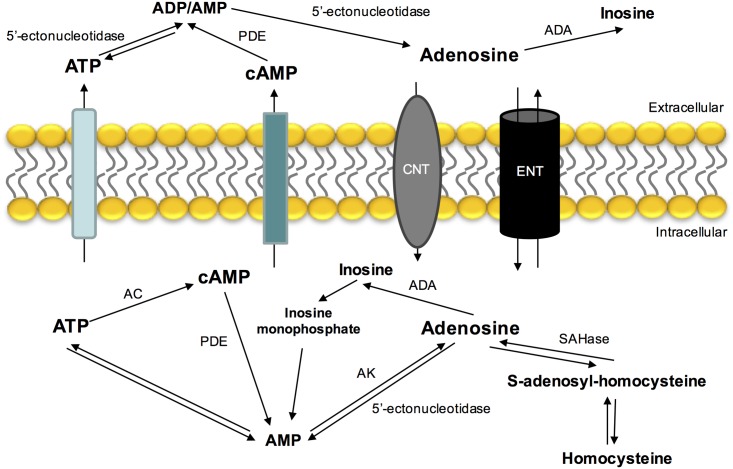
Extra- and intracellular adenosine metabolism and nucleoside transporters that contribute to its release, uptake and production. ADA, adenosine deaminase; AC, adenylyl cyclase; AK, adenosine kinase; CNT, concentrative nucleoside transporter; ENT, equilibrative nucleoside transporter; PDE, phosphodiesterase; SAHase, *S*-adenosyl homocysteine hydrolase.

Adenosine is mostly formed by the catabolism of 5′adenosine phosphates (ATP, adenosine diphosphate – ADP and adenosine monophosphate – AMP). Intracellular adenosine production is mediated by an intracellular 5′-nucleotidase that dephosphorylates AMP (Schubert et al., 1979; Zimmermann et al., 1998) or by the hydrolysis of *S*-adenosylhomocysteine by *S*-adenosylhomocysteine hydrolase (Broch and Ueland, 1980) (**Figure 1**). Extracellular adenosine comes from ATP hydrolysis via 5′ectonucleotidases (Fredholm et al., 2001; Yegutkin, 2008) and by its intracellular production and release by nucleoside transport system (for a review see Conde et al., 2009). Another source of adenosine that is present extracellularly is cyclic AMP (cAMP) that can be released by secretory cells and converted by extracellular ectophosphodiesterases in AMP and then into adenosine by 5′-ectonucleotidases (Fredholm et al., 2001).

In contrast with other neurotransmitters, adenosine is not stored in synaptic vesicles or acts exclusively on synapses. Its release and uptake occurs through nucleoside transporters, which are constituted by two families: a Na^+^ independent family and another one dependent of the same ion (Griffith and Jarvis, 1996). The Na^+^ dependent-nucleoside transport system is concentrative, carrying nucleosides against a concentration gradient. The Na^+^ independent-nucleoside transport system (equilibrative nucleoside transport system, ENT) is bi-directional and is formed by two different families (*es* and *ei*), classified based on their sensitivity to nitrobenzylthioinosine (NBTI). The *es* transport is inhibited by low nanomolar concentrations of NBTI, while *ei* transport requires micromolar concentrations to be inhibited (Griffith and Jarvis, 1996; Cass et al., 1998; Podgorska et al., 2005).

The major pathways of adenosine removal or degradation involve reactions catalyzed by two enzymes: adenosine kinase (AK) and adenosine deaminase (ADA) (Fredholm et al., 1999), which leads to the formation of inosine and AMP, respectively (Conde et al., 2009). ADA is mostly found in the intracellular space, however, it is also found in some extracellular compartments. This enzyme has relevance when adenosine concentrations are high (Arch and Newsholme, 1978) and alterations in its activity have been associated with several pathologies, such as *miastenia* gravis and diabetes mellitus (Hoshino et al., 1994; Oliveira et al., 2015).

### Adenosine Receptors

Adenosine exerts is action through four different type of adenosine receptors coupled to G proteins A_1_, A_2A_, A_2B_, and A_3_ (Conde et al., 2009). These receptors are activated by different endogenous adenosine concentrations being the affinity for adenosine: A_1_ > A_2A_ > A_2B_ > A_3_. The adenosine that is available endogenously to activate these receptors is in equilibrium with the density of adenosine receptors at the site of action to help to control the different physiological responses to this nucleotide (Conde et al., 2009).

A_1_ and A_2_ adenosine receptors have been subdivided based on their capacity of inhibiting and stimulating adenylyl cyclase and therefore, their ability to decrease and increase the cAMP levels, respectively. In fact, A_1_ and A_2_ adenosine receptors are G_i_ and G_S_-coupled receptors, respectively. The A_3_ adenosine receptors are also coupled to G_i_ proteins (Fredholm et al., 2001). However, nowadays there are some evidences that adenosine receptors may activate signaling pathways via other G proteins, for example A_1_ receptors are coupled preferentially to G_i1/2/3_, but they can also be coupled to G_o_. On the other hand, although A_2A_ and A_2B_ receptors preferentially activate G_S_ proteins, they can also activate G_olf_ and G_15/16_, and G_q_, respectively (Fredholm et al., 2001). A_3_ receptors that activate G_i/o_ proteins can also activate G_q_ (Conde et al., 2009). Apart from the activation of enzymes, the activation of G coupled proteins acts on ion channels. In addition it has been shown in hippocampal slices that A_1_ adenosine receptors activate N, P, and Q-type Ca^2+^ channels (Wu and Saggau, 1994), several types of K^+^ channels in cultured striatum mouse neurons (Trussell and Jackson, 1985) and also lead to the activation of phospholipase C (Fredholm et al., 2001). A_3_ receptors seem to mediate the same effectors than A_1_ receptors. The main second messenger involved in the activation of A_2A_ and A_2B_ receptors is cAMP, with the stimulation of these receptors originating an increase in cAMP intracellular levels, however, other actions, including mobilization of intracellular calcium, have also been described (for a review see Fredholm et al., 2001).

### Metabolic Pathways of ATP Formation and Release

Adenosine-5′-triphosphate is released from several cells in physiological conditions and/or pathophysiologically in response to hypoxia, inflammation, to mechanical stress and to some antagonists (Bodin and Burnstock, 2001; Burnstock, 2016). Classically, ATP was known to be released from nerve terminals by exocytosis, via Ca^2+^ dependent mechanisms (Zimmermann, 2016). However, apart from being released from nerve terminals it can be also released by glial cells such as astrocytes (Gordon et al., 2005) through ATP-binding-cassette transporters, surface-located hemichannels (connexin, pannexin) and plasmalemmal voltage-dependent anion channels (Zimmermann, 2016). Neuronal and glial ATP modulate postsynaptic strength though activation of postsynaptic P2X receptors (Gordon et al., 2005; Khakh and North, 2012; Pougnet et al., 2014, Neuron).

After released and exerting its action on its receptors, ATP must be removed from the synaptic clef, which is mainly performed by its breakdown by diverse types of ectonucleotidases. There are four large families of ectonucleotidase enzymes: ectonucleotide pyrophosphatase/phosphodiesterase (E-NPP), which hydrolyze ATP to AMP; ectonucleoside triphosphate diphosphohydrolase (E-NTDPase), which hydrolyze ATP to ADP or AMP; alkaline phosphatase which generate adenosine; and 5′-ectonucleotidase which hydrolyzed AMP to adenosine (Zimmermann et al., 2007; Knowles, 2011).

### ATP Receptors

Adenosine-5′-triphosphate exerts its physiological actions by the activation of its receptors that have been divided in two families: P2X ionotropic ligand-gated ion channel receptors and P2Y metabotropic G-protein-coupled receptors (Abbracchio and Burnstock, 1994; Fredholm et al., 1994). Currently are described seven subtypes of P2X receptors (P2X_1_–P2X_7_) (Fredholm et al., 1994; Ralevic and Burnstock, 1998) and eight subtypes of P2Y receptors (P2Y_1_, P2Y_2_, P2Y_4_, P2Y_6_, P2Y_11_, P2Y_12_, P2Y_13_, P2Y_14_) (Burnstock and Knight, 2004).

The P2Y receptors are divided into two subgroups. P2Y_1_, P2Y_2_, P2Y_4_, and P2Y_6_ that activate G_q_ coupled proteins and phospholipase Cβ, leading to the formation of inositol 1,4,5-trisphosphate (IP3) which increases intracellular Ca^2+^, and diacylglycerol which activates protein kinase C. In contrast, P2Y_12_, P2Y_13_, and P2Y_14_ activate G_i_, inhibiting adenylyl cyclase and decreasing intracellular cAMP levels. P2Y_11_ receptor activates both Gq and Gs, which increases both intracellular Ca^2+^ and cAMP (Zimmermann, 2016). The seven P2X receptor subunits assemble to form trimeric homomers and often some combinations of trimeric heteromers (Lewis et al., 1995; Torres et al., 1999) that mediate rapid (within 10 ms) and selective permeability to Na^+^, K^+^, and Ca^2+^ ions (Khakh and North, 2006). This is in accordance with their role as mediators of ATP action as neurotransmitter or neuromodulator of fast synaptic transmission (Khakh and North, 2012; Boué-Grabot and Pankratov, 2017) in both central and peripheral nervous systems. These P2X receptors can be located at pre-synaptic level (facilitating neurotransmitter release) and at post-synaptic level modulating synapse strength (for a review see North, 2016). In contrast, P2Y receptors, which involves coupling to G proteins and second-messenger systems present a slower onset of response (less than 100 ms) to ATP (for a review Ralevic and Burnstock, 1998).

## Role of Purines on the Hypoxic Response in the Carotid Body

### The Carotid Bodies

The carotid bodies (CB) are paired chemoreceptors located in the bifurcation of the common carotid artery that are involved in the sensing of changes in arterial blood gasses such as hypoxia, hypercapnia, and acidosis. These stimuli generate action potentials at the CB sensitive nerve, the CSN, that are integrated in the brainstem to induce cardiorespiratory responses, to normalize blood gasses via hyperventilation (Gonzalez et al., 1994), and to regulate blood pressure and cardiac performance via activation of the sympathetic nervous system (Marshall, 1994).

The CB is organized into glomeruli, which are clusters of cells in close contact with a profuse network of capillaries and connective tissue. Each glomerulus contains chemoreceptor cells, also known as glomus or type I cells, which are derived of the neural crest and that are synaptically connected with the sensory nerve endings of the CSN (Gonzalez et al., 1994). Chemoreceptor cells contain several classical neurotransmitters as catecholamines (dopamine and norepinephrine), serotonin, acetylcholine, neuropeptides (substance P and enkephalins), but also contain ATP and adenosine (Gonzalez et al., 1994; Zhang et al., 2000; Rong et al., 2003; Buttigieg and Nurse, 2004; Conde and Monteiro, 2004; Conde et al., 2012a). Chemoreceptor/type I cells are enclosed by type II cells or sustentacular cells. It has been proposed that type II cells exhibit properties of stem cells that in response to hypoxia can proliferate and differentiate into new type I cells (Pardal et al., 2007).

### Adenosine and ATP Receptors in the Carotid Body

The CB possesses receptors for both adenosine and ATP. The presence of A_1_ receptors at the CB is not consensual. Rocher et al. (1999) described that A_1_ receptors are present in rabbit CB chemoreceptor cells, since A_1_ antagonists, DPCPX (10 μM) and 8-cyclopentyl-1,3-dimethylxantine (0.1 μM) prevented the inhibitory action of adenosine on L-type Ca^2+^ currents and on the release of catecholamines. A_1_ receptors were also detected in the whole rat CB structure (Bairam et al., 2009). However, other authors described that A_1_ receptors are absent in rat CB chemoreceptor cells (Gauda et al., 2000; Kobayashi et al., 2000) being present in the petrosal ganglion neurons that also express tyrosine hydroxylase (TH) mRNA (Gauda, 2002). The discrepancies described between the existence of A_1_ receptors in the CB could be due to different receptor localization in the CB structures and due to the different species studied.

Among the different adenosine receptor subtypes, A_2A_ and A_2B_ receptors were the main receptors localized in the CB chemoreceptor cells. A_2A_ mRNA expression is developmentally regulated in the CB (Gauda et al., 2000) as it occurs with TH and dopamine D_2_-receptors mRNAs (Gauda et al., 1996). The expression of A_2A_ receptors and their colocalization with TH in rat CB chemoreceptor cells have been demonstrated by immunocytochemistry (Gauda et al., 2000; Kobayashi et al., 2000). A_2B_ receptors were also present in rat CB chemoreceptor cells (Conde et al., 2006), as they colocalize with TH. Moreover, it was demonstrated, through the pharmacological decomposition of the effects of caffeine, a non-selective antagonist of adenosine receptors, on the CSN action potential frequency of discharge, that A_2A_ are also present post-synaptically on the CSN (Conde et al., 2006).

The expression of A_3_ adenosine receptors was not detected in CB chemoreceptor cells (Kobayashi et al., 2000).

Regarding ATP receptors, McQueen and Ribeiro (1983) proposed for the first time the presence of P2 receptors in the cat CB based on experimental data obtained using the ATP analog, the αβ-methylene ATP. Later, the same authors concluded that P2X receptors were present in the rat CB, since P2X agonists activated the carotid chemoreceptor afferents (McQueen et al., 1998). In co-cultures of rat type I cells and petrosal ganglion cells P2X_2_ receptors were present in the afferent terminals surrounding clusters of chemoreceptor cells, but not in chemoreceptor cells themselves, suggesting a post-synaptic localization (Zhang et al., 2000). One year later, a study from the same group showed that P2X_3_ receptors were also present in chemoafferent CB neurons and that P2X_2_ and P2X_3_ colocalize in synaptic terminals opposed to chemoreceptors cells, forming a heterodimeric receptor (Prasad et al., 2001). In addition, in co-cultures of rat CB and glossopharyngeal neurons it has also been showed that glossopharyngeal neurons expressed at least four different subtypes of P2X receptors (P2X_2_, P2X_3_, P2X_4_, and P2X_7_) (Campanucci et al., 2006).

Apart from the presence of P2X ATP receptors, P2Y receptors were also described in the CB (Xu et al., 2003, 2005). In rat CB dissociated cells it has been shown that ATP triggers a transient rise in intracellular Ca^2+^ in type II cells, but not in type I cells, and that P2Y_2_ receptors are localized in type II cells (Xu et al., 2003). Moreover, Xu et al. (2005) described the presence of P2Y_1_ receptors in the CB since it was observed in CB type I dissociated cells that ATP suppressed the hypoxia-induced intracellular Ca^2+^ rise via the activation of P2Y_1_ receptors being the order of purinoreceptor agonist potency in inhibiting the hypoxia response in agreement with the involvement of P2Y_1_ receptors.

### Adenosine Effects on Ventilation and on Carotid Sinus Nerve Activity

Adenosine increases ventilation in several species, and this effect was attributed to the activation of CB chemoreceptors. Studies performed in humans showed that adenosine increases ventilation in a dose-dependent manner, an effect that is also dependent on the proximity of adenosine administration to the CB (Watt and Routledge, 1985; Watt et al., 1987), meaning that the effect is as higher as closer is adenosine administration from the CB. Consistent with the effect of adenosine in modulating ventilation via CB chemoreceptors, intra-arterially administration of adenosine in dog and cats showed that adenosine does not cross the blood brain barrier (Berne et al., 1974). Moreover, the effect of adenosine and its antagonists on ventilation in response to hypoxia was suggested to involve a mechanism of peripheral chemoreception, the CB, rather than effects on CNS (Maxwell et al., 1986, 1987). In humans, the intravenous infusion of adenosine, that is commercially available as antiarrhythmic, induces chest discomfort, hyperventilation and dyspnea, being these effects attributed to CB chemoreceptors activation (Watt and Routledge, 1985; Maxwell et al., 1986, 1987; Watt et al., 1987; Uematsu et al., 2000).

In the rat, intracarotid administration of adenosine and its analogs increased in a dose-dependent manner ventilation an effect abolished after CSN section (Monteiro and Ribeiro, 1987). This excitatory effect of adenosine on ventilation was mediated by A_2_ receptors (Monteiro and Ribeiro, 1987; Ribeiro and Monteiro, 1991), and it seems that A_2A_ are responsible, at least in part, by this effect, since CGS21680, an A_2A_ selective agonist, increased ventilation in rats by 31% (Conde et al., 2009). A work performed in rhesus monkeys also supports the excitatory effect of adenosine on ventilation (Howell and Landrum, 1995). In this work, it was described that caffeine, a non-selective adenosine receptor antagonist, attenuated hypoxia-induced increases in ventilation when animals were exposed to 10% O_2_ (Howell and Landrum, 1995). Furthermore, intracarotid administration of erythro-9-(2-hydroxy-3-nonyl) adenine (EHNA) and dipyridamole, inhibitors of adenosine deamination and uptake, respectively, leading to an increase in endogenous adenosine, emulated the excitatory effect of exogenous adenosine on ventilation (Monteiro and Ribeiro, 1989).

Besides, the demonstration of the role of adenosine on modulating ventilation via the CB, in McQueen and Ribeiro (1981) described for the first time that adenosine can stimulate the CSN chemosensory activity. This effect of adenosine on CSN chemosensory activity was mimicked by adenosine analogs and inhibited by theophylline and 8-phenyltheophylline, suggesting the presence and involvement of A_2_ receptors (McQueen and Ribeiro, 1983, 1986). *In vitro* experiments in cats and rats corroborate these findings, since it was demonstrated that adenosine augments chemoreceptor discharge (Runold et al., 1990; Vandier et al., 1999), an effect that is dose dependent (Runold et al., 1990). Furthermore, McQueen and Ribeiro (1986) also described that intracarotid administration of 8-phenyltheophylline, an adenosine receptor antagonist, in the cat reduced the CB chemoreceptor response to hypoxia (10% O_2_), which could indicate that adenosine released by the CB during hypoxia acts directly on nerve endings or as a modulator. These findings were supported by a previous work from the same group, where it was described an increase in CB chemoreceptor discharge in cat under normoxic conditions when adenosine uptake is inhibited by dipyridamole, suggesting that increases in the levels of endogenous adenosine cause chemoexcitation (McQueen and Ribeiro, 1983). In 2006, our group described that the CSN chemosensory activity elicited by hypoxia (5% O_2_) is modulated by adenosine, an effect that is mediated by its action on both A_2B_ presynaptic receptors (25%) present in CB type I cells and A_2A_ postsynaptic receptors (30%) in CSN nerve endings (Conde et al., 2006).

### Effects of ATP on Ventilation and Carotid Sinus Nerve Activity

The first evidence that ATP could affect ventilation was described by Anichkov and Belen’kii (1963), in a work that showed an increase in ventilation when ATP was administrated into the carotid bifurcation of decerebrated cats. Later, an autoradiographic study described the presence of ATP in the mouse CB (Kobayashi, 1976) and, nucleoside triphosphatase activity was detected in cat CB homogenates (Starlinger, 1982). Reyes et al. (2007) demonstrated in cats a dose-dependent excitatory effect of ATP on ventilation that was mediated through P2 receptors since the effect of ATP on ventilation was suppressed by suramin. Also, ATP and P2X_2_ receptors are involved in the ventilatory responses to hypoxia mediated by the CB, since mice deficient in P2X_2_ receptors exhibited a prominent diminished ventilatory response to hypoxia, being this effect inversely correlated with hypoxia intensity, meaning that the decrease in ventilation is higher when the PaO_2_ decreases (Rong et al., 2003). In contrast, mice deficient in P2X_3_ receptors subunit showed a response to hypoxia comparable with the response of wild-type animals (Rong et al., 2003), suggesting that the P2X_3_ receptors that are also present in the CB do not mediate the ventilatory responses to hypoxia.

The results of ATP on ventilation are also consistent with the effect of ATP on CSN chemosensory activity. In the early 1950s, Jarisch et al. (1952) described an increase in CSN chemoreceptor discharge following an intracarotid administration of ATP. This excitatory effect of ATP on CSN chemoreceptor activity was also described by other *in vivo* and *in vitro* studies (Dontas, 1955; McQueen and Ribeiro, 1983; Ribeiro and McQueen, 1984; Spergel and Lahiri, 1993). Moreover, it was showed that this effect of ATP on CSN activity was dose-dependent (McQueen and Ribeiro, 1983; Alcayaga et al., 2000; Reyes et al., 2007; Soto et al., 2010) and due to ATP itself and not to its degradation into adenosine since the ATP agonists, βγ-methylene ATP promoted increases in the CSN chemoreceptor activity in cats (McQueen and Ribeiro, 1983; Reyes et al., 2007) and αβ-methylene ATP increased CSN discharges in rats (McQueen et al., 1998) and mice (Rong et al., 2003). Additionally, P2X receptor agonists induced rapid cardiorespiratory reflexes in anesthetized rat, suggesting the presence of this receptors in the rat CB (McQueen et al., 1998). These findings were supported by a work by Colin Nurse group (Zhang et al., 2000). They showed in a co-culture model of rat type I cell clusters and petrosal neurons that the application of suramin partially inhibited hypoxia-induced postsynaptic responses recorded in petrosal neurons (Zhang et al., 2000). In addition, both P2X_2_ and P2X_3_ receptor subunits were immunolocalized with petrosal afferent terminals in the rat CB (Zhang et al., 2000; Prasad et al., 2001). Furthermore, Rong et al. (2003) not only showed that P2X_2_ subunit are involved in the CB-mediated ventilatory responses to hypoxia, as herein described, but also showed a substantial decrease in the CSN responses to hypoxia in an *in vitro* CB-CSN preparation from mice deficient in P2X_2_ subunits. Therefore, is now accepted that ATP is an excitatory neurotransmitter at the synapse between the CB and the CSN and that is involved in the CB response to hypoxia. However, the contribution of ATP for the hypoxic signaling in the CB is dependent on hypoxia intensity, with ATP having a more pronounced role in the response to high intensity hypoxias and adenosine with moderate hypoxias (Conde et al., 2012a), suggesting that the response to hypoxia in the CB are related with alterations in the ATP/adenosine metabolism.

While the effect of ATP in CB response to hypoxia is consensual, some controversy exists on the effect of ATP in fixing basal CSN activity. Zhang et al. (2000) described that suramin inhibited CSN basal activity. In contrast, our group reported that suramin did not modify CSN basal activity, which suggests that ATP is not the mediator involved in fixing the steady basal CSN chemosensory activity in adult rat (Conde et al., 2012a). These discrepancies could be related with developmental differences since the experiments performed by Zhang et al. (2000) were performed in postnatal 7- to 14-day-old rat pups. In fact, Niane et al. (2011) described a decrease in spontaneous CSN activity (80%) in newborn rats, an effect was constant across ages (4- to 21-day-old rats). However, previous studies of the same authors (Donnelly and Doyle, 1994) have shown that both basal and hypoxia-induced CSN activity increases with age. Additionally, Niane et al. (2011) showed by the use of a specific P2X_3_ antagonist, A-317491, that in the CB from newborn rats, the P2X_3_ receptor subunit plays a major role in the regulation of breathing under basal and hypoxic conditions, which is in contradiction with the results from Rong et al. (2003) in the mice. However, in both Zhang et al. (2000) and Niane et al. (2011) suramin was insufficient to fully promote inhibition of ventilation and the CSN chemosensory response to hypoxia suggesting that other excitatory co-transmitters are also involved (Fitzgerald, 2000; Iturriaga and Alcayaga, 2004; Zapata, 2007; Nurse, 2010; Conde et al., 2012a). Several authors have proposed the co-release of ATP-Acetylcholine, since the application of a mixture of nicotinic and purinergic antagonists completely suppress the CSN response to hypoxia (Zhang et al., 2000; Varas et al., 2003), however, Reyes et al. (2007) described that the perfusion of CB excised from cats with a mixture of nicotinic and purinergic antagonists was not able to eliminate the chemosensory response to hypoxia stimulation. Therefore, the hypothesis of the co-signaling of ATP-Acetylcholine in the CB remains controversial while the hypothesis of ATP-adenosine co-transmission gained many supporters.

### ATP and Adenosine Release from Carotid Body

The first evidence for CB ATP release was a report describing a decrease in ATP content in the cat CB incubated with moderate hypoxia during 5 min or with cyanide, an inhibitor of the mitochondrial electron transport that induced a decrease in ATP and an increase in AMP content (Obeso et al., 1985, 1989). Also, rabbit CBs superfused during 15 min with cyanide or antimycin exhibit reduced ATP levels (Verna et al., 1990). In contrast with these results, it was observed that CB ATP levels were unchanged: (1) in cats exposed to hypoxia or hypercapnia (Acker and Starlinger, 1984); (2) in cat CBs incubated in the presence of dinitrophenol (Obeso et al., 1989), an uncoupler of oxidative phosphorylation that targets the mitochondria; (3) and in rabbit CBs superfused during 4–30 min with 10% O_2_-equilibrated Krebs–Henseleit solution (Verna et al., 1990). Buttigieg and Nurse (2004), described that acute hypoxia evoked an increase in extracellular ATP in the whole CB, an effect that was inhibited by L-type Ca^2+^ channel blockers. In addition, observations from our group showed that adult rat CBs incubated in Tyrode solution equilibrated with different O_2_ concentrations released higher concentrations of ATP when exposed to hypoxia (2% O_2_ and 10% O_2_) than when exposed to 20% O_2_ and 95% O_2_ (Conde and Monteiro, 2006). More recently, we showed that the release of ATP from rat CB is proportional with hypoxia intensity (Conde et al., 2012a) and that the increase in ATP release induced by hypoxia was completely prevented by removal of extracellular calcium and by a calcium chelating agent, suggesting that ATP released during hypoxia comes from a vesicular source through exocytosis (Conde and Monteiro, 2006; Conde et al., 2012a). Therefore, the signaling cascade between hypoxic signal and the release of ATP would be: (1) detection of hypoxia by an O_2_ sensor (molecular identity unknown), (2) closure of K^+^ channels, (3) opening of Ca^2+^ channels, (4) increase in intracellular calcium, (5) release of ATP by exocytosis (Gonzalez et al., 1994, 2010; Conde et al., 2012a).

Adenosine is also released from the CB. Our group showed that in adult rat CB adenosine is released in normoxic conditions, and its release augments in response to 10 and 30 min of moderate hypoxia (10% O_2_) (Conde and Monteiro, 2004), but is not modified by hyperoxic exposure (95% O_2_) (Conde and Monteiro, 2006). These experiments were performed under incubation of adenosine deaminase due to the short life-time of adenosine and to avoid its degradation. In contrast, the CB adenosine content was drastically reduced after 30 min of hypoxic exposure (Conde and Monteiro, 2004). Also, we have showed that approximately 40% of adenosine present extracellularly in the CB came from extracellular ATP degradation, both under normoxic and hypoxic conditions and that low pO(2) triggers adenosine efflux through the activation of NBTI-sensitive ENT. This effect was only apparent in hypoxia and when adenosine extracellular concentrations were reduced by the blockade of ecto-5′-nucleotidase (Conde and Monteiro, 2004).

Although we have showed that both ATP extracellular catabolism as well as release of adenosine *per se* through an NBTI-sensible ENT can account to the amount of adenosine present in the CB-CSN synapse, we cannot exclude another sources of extracellular adenosine, as cAMP. Even though several studies reported the role of cAMP in CB chemotransduction and/or chemotransmission (Nunes et al., 2014), the contribution of extracellular cAMP to extracellular adenosine has never been investigated in the CB. Additionally, other mechanisms, such as inhibition of *S*-adenosylhomocysteine and adenosine deaminase, could be involved in adenosine production and release by the CB in normoxia and hypoxia.

Additionally, our group demonstrated that adenosine is preferentially released in response to moderate hypoxia (10% O_2_) than in response to higher hypoxic intensities (2% O_2_ and 5% O_2_), while CB ATP release had a more pronounced role during high intensity hypoxias (Conde et al., 2012a). These findings were corroborated by electrophysiological data showing that ZM241385, in a concentration that block A_2_ adenosine receptors (A_2A_ and A_2B_, 300 nM), inhibits CSN chemosensory activity with higher efficacy in moderate hypoxia than in intense hypoxia (Conde et al., 2012a). Furthermore, it was also shown that during a high-intense hypoxia the main origin of extracellular adenosine is ATP catabolism, whereas at moderate hypoxia the main source of adenosine is its release *per se* by the ENT (Conde et al., 2012a). All together these findings showed that adenosine acting on the CB via A_2A_ and A_2B_ receptors together with ATP acting on P2X receptors are key neurotransmitters involved in hypoxic CB chemotransduction, depending the contribution of each neurotransmitter on the hypoxia intensity.

As previously described, the conversion of ATP to adenosine requires both membrane bound E-NTDPase and 5′-ectonucleotidases and, it was recently described that E-NTDPase2,3 are expressed prominently in the periphery of CB type I cells in the vicinity with CSN endings and that 5′-ectonucleotidase (CD73) is expressed in both types I and II cells (Salman et al., 2017). Holmes et al. (2017) recently showed that the inhibition of CD73 decreased the basal CSN activity and attenuated the responses to hypoxia. These authors also described that the *in vivo* inhibition of CD73 with AOPCP, blunted the hypoxic ventilatory response and reduced the elevation in the heart rate induced by hypoxia, showing that CD73 regulates peripheral chemoreceptor activity and the cardiorespiratory responses to hypoxia (Holmes et al., 2017). Additionally, it has been shown that under chronic hypobaric hypoxia an upregulation of E-NTDPase3 and CD73 was observed, while E-NTDPase2 was downregulated, suggesting that this differential regulation leading to alterations in purinergic adenosine and P2 receptors signaling, may contribute to CB plasticity during chronic hypoxia (Salman et al., 2017). These results together suggest a hypoxic modulation of purines metabolism at the CB that control the contribution of adenosine and ATP in CB chemotransduction both in basal conditions as well as in the responses to acute and chronic hypoxia.

### Cellular Actions of Adenosine in the Carotid Body

At the moment, it is accepted that the chemoexcitatory effect of adenosine at the CB involves the activation of adenosine receptors and consequently the activation of cellular pathways activated by G-coupled receptors as well as alterations in cAMP and Ca^2+^ intracellular levels, cell depolarization among other events. There is a consensus that adenosine and its analogs increase cAMP levels in the rat (Monteiro et al., 1996; Conde et al., 2008) and rabbit CB (Chen et al., 1997). Hypoxia also induced an increase in cAMP in CB type I cells (Pérez-García et al., 1990; Wang et al., 1991) an effect that was potentiated by adenosine, since dipyridamole, an inhibitor of adenosine uptake, increased cAMP content in rabbit CB superfused with 5% O_2_. This effect was blocked by A_2_ adenosine receptors antagonists (Chen et al., 1997) meaning that A_2_ receptors mediated the increase in cAMP produced by hypoxia. More recently, it was shown that this increase in cAMP levels evoked by adenosine is mostly mediated by the activation of A_2B_ adenosine receptors (Conde et al., 2008).

Another effector of cAMP is K^+^ channels, and in fact K^+^ channels are known to be modulated by the increase in cAMP levels induced by adenosine at the CB (López-López et al., 1993). López-López et al. (1993) showed that the application of a cAMP analog, dibutyryl cAMP, in isolated rabbit type I cells, decreased the amplitude of 4-aminopyridine-sensitive K^+^ currents, an effect that is voltage independent. In contrast, Hatton and Peers (1996) demonstrated that dibutyryl cAMP (5 mM) and 8-bromo-cAMP (2 mM) were unable to modify K^+^ current amplitudes in isolated rat type I cells. These discrepant results could be related with animal’s age and with differences between the electrophysiological properties and responses to hypoxia of rabbit and rat type I cells (Peers and Buckler, 1995). Furthermore, Vandier et al. (1999) showed that adenosine decreased the amplitude of 4-aminopyridine-sensitive K^+^ currents in isolated rat type I cells, an effect that is voltage independent and mainly Ca^2+^ dependent. However, a small but significant component of the current blocked by adenosine was Ca^2+^ dependent (Vandier et al., 1999). Additionally, in isolated rabbit type I cells, adenosine inhibits L-type Ca^2+^ channels and the release of catecholamines induced by hypoxia, an effect that was described to be mediated by A_1_ adenosine receptors, since A_1_ agonists and antagonists are capable of modulate Ca^2+^ currents (Rocher et al., 1999). Kobayashi et al. (2000) also described that adenosine inhibits voltage-dependent Ca^2+^ currents in isolated rat type I cells. However, this effect was attributed to A_2A_ adenosine receptors, since ZM241385, in a concentration that is specific for A_2A_ receptors (10 nM), abolished the effect of adenosine on Ca^2+^ currents (Kobayashi et al., 2000). This discrepancies as previously discussed could be attributed to differences between species. Additionally, adenosine attenuated the increase in intracellular Ca^2+^ evoked by hypoxia without changing the intracellular Ca^2+^ in cells exposed to normoxia (Kobayashi et al., 2000). In contrast, Xu et al. (2006) observed that adenosine via A_2A_ receptors elicited a rise in intracellular Ca^2+^. The authors also described that this effect of adenosine on intracellular Ca^2+^ occurs through the action of adenosine on adenylate cyclase and protein kinase A pathways, which inhibits the TWIK-related acid-sensitive K^+^-1 (TASK-1) channels, leading to depolarization and, therefore to Ca^2+^ entry via voltage-gated Ca^2+^ channels (VGCC) (Xu et al., 2006; Tse et al., 2012). However, the increase in the intracellular Ca^2+^ observed by Xu et al. (2006) may be insufficient to evoke the release of neurotransmitters, since it is much smaller than the increase that evokes the release of catecholamines from the rat CB (Vicario et al., 2000). Moreover, the block of A_2A_ adenosine receptors with SCH58261, a selective antagonist of A_2A_ receptors in the concentration used (5 nM), decreases hypoxia-evoked receptor potentials in rat type I cells (Nurse and Piskuric, 2013). The role of adenosine in modulating CB cells function in chronic hypoxia was also highlighted by the fact that the exposure of rat CB cultures to chronic hypoxia (2% O_2_, 24 h) induced an augment in adenosine-evoked increases intracellular Ca^2+^ transients and catecholamine secretion from CB type I cells, an effect that is mediated by A_2B_ receptors (Livermore and Nurse, 2013). This pathway could contribute to CB sensitization during ventilatory acclimatization to hypoxia in animals and humans exposed to chronic hypoxia *in vivo* (Conde et al., 2009; Teppema and Dahan, 2010).

**FIGURE 2:**
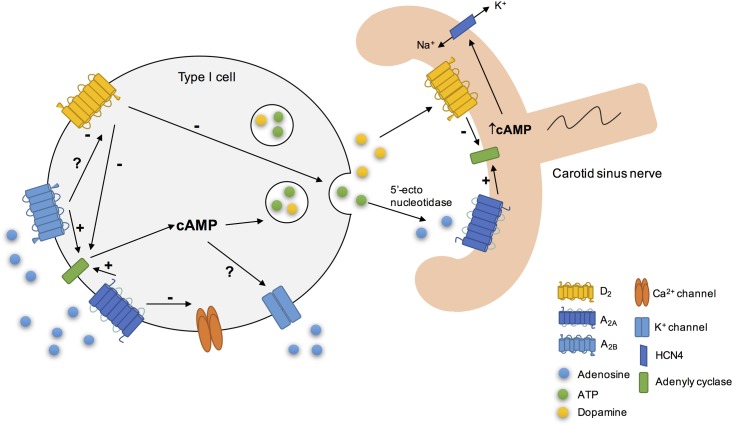
Schematic representation of some of adenosine cellular actions on rat carotid body (CB). Adenosine increased cAMP content in type I cells via A_2A_ and A_2B_ action on adenylyl cyclase, leading to the release of neurotransmitters, as catecholamines (Conde et al., 2008). Additionally, its action on these receptors could modulate K^+^ currents (López-López et al., 1993) for example by decreasing the amplitude of K^+^ currents (Vandier et al., 1999) and inhibit the voltage-dependent Ca^2+^ currents in type I cells. During hypoxia, adenosine released *per se* through the equilibrative nucleoside transport system or generated by the extracellular breakdown of ATP by 5′-ectonucleotidases (Conde and Monteiro, 2004; Conde et al., 2012a; Salman et al., 2017), acts postsynaptically on A_2A_ receptors, leading to adenylyl cyclase activation and to an increase in cAMP, which stimulates HCN4-containing non-selective cation channels that mediate *I*_h_, leading to an increase in membrane excitability. In contrast, dopamine exerts the opposite effect, leading to a decrease in petrosal membrane excitability (Zhang et al., 2017).

Adenosine also acts as a neuromodulator in CB chemoreceptor cells since it acts to modulate the release of other neurotransmitters. It has been showed that adenosine is involved in the release of catecholamines through the antagonist interaction between A_2B_ and dopamine D_2_ receptors (Conde et al., 2008) (**Figure 2**). This interaction between A_2B_ and D_2_ receptors in CB chemoreceptors cells is evident at adenylyl cyclase level, since D_2_ agonists inhibited cAMP production in CB, an effect that is prevented by an A_2B_ receptor antagonist and occurs in basal conditions as well as hypoxia (Conde et al., 2008). However, an antagonistic interaction at the A_2B_-D_2_ receptor level, similar to that described in the CNS for A_2A_-D_2_ receptors (Fuxe et al., 2007) cannot be excluded. These results are in agreement with a previous work from Monteiro and Ribeiro (2000) that described an enhancement of the inhibitory effect of dopamine on ventilation induced by the intracarotid infusion of adenosine. Regarding the interactions between adenosine and dopamine at postsynaptic level, a recent work from Zhang et al. (2017) showed in co-culture of rat CB type I cells and sensory petrosal neurons that adenosine increases a hyperpolarization-activated cyclic nucleotide-gated (HCN) cation current *I*_h_ in chemosensory petrosal neurons through A_2A_ receptors, whereas dopamine had the opposite effect through D_2_ receptors. The effect of adenosine on HCN cation current *I*_h_ seems to involve the activation of adenylyl cyclase and the increase in intracellular cAMP that in turn activates HCN4-containing non-selective cation channels that mediate *I*_h_ (Zhang et al., 2017). Moreover, the authors obtained evidence for a presynaptic role for adenosine acting via A_2A_ receptors during chemotransduction, since SCH58261 inhibited both hypoxia-induced presynaptic receptor potential and postsynaptic petrosal response (Zhang et al., 2017).

### Cellular Actions of ATP in the Carotid Body

During hypoxia it was observed that rat chemoreceptors cells depolarized due to the closure of TASK1/3 background K^+^ channels (Buckler, 2015) leading to the activation of extracellular Ca^2+^ entry via VGCCs triggering the release of several neurotransmitters from chemoreceptor cells, including ATP and adenosine (Buttigieg and Nurse, 2004; Conde and Monteiro, 2004; Conde et al., 2012a). In addition, it has been shown that ATP released by type I cells can induce a rise in intracellular Ca^2+^ in isolated type II cells (Xu et al., 2003; Tse et al., 2012), since the application of different purinoceptor agonists on dissociated cell cultures of types I and II CB showed that ATP acts on type II cells via P2Y_2_ receptors subtype (Xu et al., 2003). Later, it was described that P2Y_2_ receptors activation on type II cells lead to an increase in intracellular Ca^2+^ levels and to a prolonged membrane depolarization due to the opening of large-pore, pannexin-1 channels (Zhang et al., 2012). Moreover, it was also showed in co-cultures of dissociated CB cells and petrosal neurons that the selective activation of P2Y_2_ receptors on type II cells can lead to ATP release through pannexin-1 channels, an effect that was reversibly inhibited by Panx-1 selective blocker, carbenoxolone (Zhang et al., 2012). These results lead to the authors to propose that CB type II cells may function as an ATP signal amplifier and therefore contribute to chemoexcitation through the mechanism of ATP-induced ATP release (Zhang et al., 2012).

More recently, Murali and Nurse (2016) suggested that the crosstalk between CB type I cells and type II cells during chemotransduction is mediated by purinergic signaling. In isolated rat chemoreceptor clusters, it was observed a delayed intracellular Ca^2+^ elevations in nearby type II cells that was promoted by type I cell depolarization induced by hypoxia, hypercapnia or high K^+^, an effect blocked by the P2Y_2_ antagonist suramin (Murali and Nurse, 2016). In contrast, when P2Y_2_ receptors in type II cells were stimulated induced a delayed, secondary intracellular Ca^2+^ elevations in nearby type I cells, an effect that was blocked by inhibitors of pannexin-1 channels as well as by inhibitor of A_2A_ adenosine receptors and 5′-ectonucleotidase (Murali and Nurse, 2016). Therefore, this work demonstrated that the ATP released through pannexin-1 channels in type II cells and that is catabolized extracellularly by 5′-ectonucleotidase into adenosine is the principal source of adenosine mediating the crosstalk between types I and II cells (Murali and Nurse, 2016). The adenosine that is produced extracellularly then can stimulate A_2A_ receptors that are present in type I cells inducing the increase in intracellular Ca^2+^ (Xu et al., 2006; Tse et al., 2012; Nurse and Piskuric, 2013). On the postsynaptic side at the CSN nerve endings, adenosine could increase the CSN discharge through the activation of A_2A_ adenosine receptors on afferent nerve terminals (Conde et al., 2012a). However, since it was observed that even in the presence of AOPCP a residual Ca^2+^ response in type I cells persists, it cannot be excluded the possibility of type II cells via pannexin-1 channels directly release small amounts of adenosine (Murali and Nurse, 2016).

**FIGURE 3:**
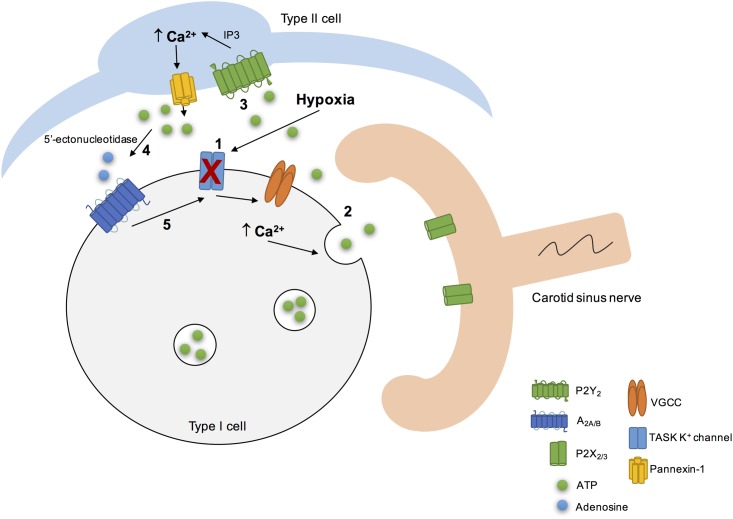
Schematic representation of the CB “tripartite” synapse model proposed by Nurse and collaborators. Hypoxia induced type I cell depolarization through the inhibition of TASK1/3 K^+^ channels (1), leading to Ca^2+^ entry via voltage-gated Ca^2+^ channels (VGCC) and to ATP release (2). ATP excites postsynaptic P2X_2/3_ receptors on petrosal nerve terminal. ATP can also stimulate P2Y_2_ receptors in adjacent type II cells (3), leading to the Ca^2+^ release from intracellular stores via inositol triphosphate (IP3) signaling pathways and opening of pannexin-1 channels. This results in ATP release that could be break down by extracellular 5’ectonucleotidase into adenosine (4) (Conde et al., 2012a; Salman et al., 2017). Adenosine stimulates A_2A_ adenosine receptors in type I cells, leading to the inhibition of TASK1/3 K^+^ channels, that enhance type I cell depolarization (5) (Xu et al., 2005) and, therefore ATP release. It is not represented but hypoxia stimulates adenosine release *per se* from type I cells (Conde and Monteiro, 2004) and high levels of ATP could inhibit pannexin-1 channels in type II cells and inhibit the chemoreceptor function via P2Y_1_ receptors, through a negative feedback mechanism. Adapted from Zhang et al. (2012), Nurse (2014), Murali and Nurse (2016).

Adenosine-5′-triphosphate itself could also regulate its own extracellular levels at the synapse. High levels of extracellular ATP could induce a negative feedback loop to inhibit pannexin-1 channels thereby regulating ATP release from type II cells (Dubyak, 2009). Additionally, high extracellular levels of ATP did not affect the resting intracellular Ca^2+^ (Xu et al., 2003) but strongly inhibited the hypoxia-induced elevation in intracellular Ca^2+^ in type I cells via a negative feedback mechanism involving P2Y_1_ receptors (Xu et al., 2005; Tse et al., 2012). The mechanism behind this effect involved the closure of background conductance(s) other than TASK-like K^+^, maxi-K or Na^+^ channels (Xu et al., 2005; Tse et al., 2012). However, this negative feedback promoted by ATP on type I cells via P2Y_1_ could be counteracted by the positive feedback action of adenosine on the presynaptic and/or postsynaptic side. All these findings about the purinergic signaling in the rat CB leads Colin Nurse group to propose a model of the CB “tripartite” synapse (**Figure 3**) (Zhang et al., 2012; Piskuric and Nurse, 2013; Nurse, 2014; Murali and Nurse, 2016).

## Role of Purines in Carotid Body-Mediated Pathologies

**FIGURE 4:**
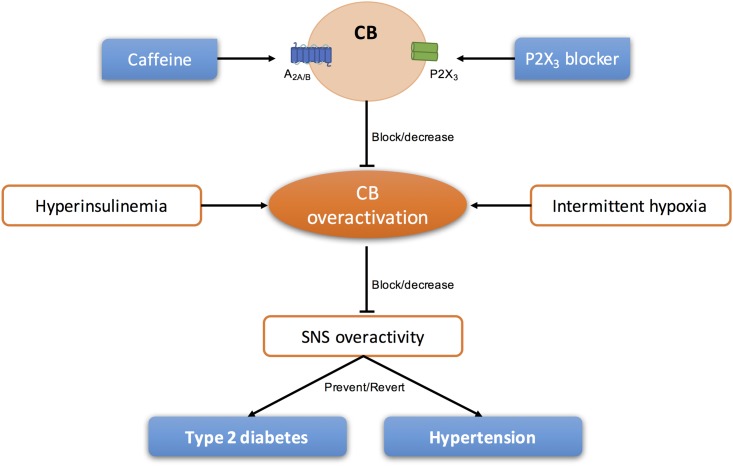
Schematic representation of the modulation of purinergic systems to block/decrease the overactivation of CB present in sympathetic-mediated diseases, as type 2 diabetes and essential hypertension.

In the last years, several literature was published defending the idea that the CB could be a therapeutic target for the treatment of sympathetically mediated diseases, as CB activity seems to be increased and involved in the pathogenesis of these diseases (Paton et al., 2013; Iturriaga, 2017). Animal and human studies have suggested the use of unilateral and/or bilateral CB ablation for the treatment of essential hypertension and heart failure (Abdala et al., 2012; Niewinski et al., 2013, 2017; Fudim et al., 2015; Narkiewicz et al., 2016). However, knowing that the surgical resection of the CSN is prone to cause side effects (for a review see Iturriaga, 2017; Sacramento et al., 2017b) other approaches that do not permanently restrict carotid body (CB) function may be more appropriate in the long term. Therefore, modulation of purines levels and/or action could be a strategy to treat pathologies associated with CB dysfunction (**Figure 4**).

### Type 2 Diabetes

In 2013, our group demonstrated for the first time that CB regulates peripheral insulin sensitivity and that CB overactivation is involved in the genesis of hypertension and insulin resistance induced by hypercaloric diets (Ribeiro et al., 2013), an effect that seems to be mediated by a sympathetic nervous system overactivation (Ribeiro et al., 2013; Sacramento et al., 2017b). Moreover, we also showed that insulin triggers CB activation, suggesting that hyperinsulinemia may be one of the stimulus responsible for CB overactivation leading to sympathetic nervous system overactivity that is associated with metabolic disturbances, such as type 2 diabetes (Ribeiro et al., 2013). In the same work, we described that bilateral CSN resection prevents the development of dysmetabolic changes induced by hypercaloric diets (Ribeiro et al., 2013) and more recently we have demonstrated that bilateral, but not unilateral CSN resection, restores insulin sensitivity and glucose homeostasis in prediabetes and type 2 diabetic rats (Sacramento et al., 2017a,b) suggesting that CB modulation could be used as a therapeutic approach.

Knowing that adenosine is one of the key neurotransmitters in the CB (Conde and Monteiro, 2004; Conde et al., 2012a) and that caffeine decreases CB activity acutely (Conde et al., 2006) and chronically (Conde et al., 2012c) by approximately 60%, it is expected that the overactivation of the CB seen in hypercaloric animal models could be decreased with long-term administration of caffeine. In fact, our group described that chronic caffeine intake prevents and reverted the increase in blood pressure and insulin resistance in hypercaloric animal models of prediabetes (Conde et al., 2012b). Additionally, epidemiological studies described that chronic caffeine consumption is associated with a significant lower risk of diabetes (van Dam and Hu, 2005; Ding et al., 2014). Therefore, it remains to prove that CB overactivation in hypercaloric animal models of diabetes is decreased in the presence of long-term caffeine treatment as well as the link caffeine-decreased CB activity- decreased sympathetic nervous system activity.

### Obstructive Sleep Apnea

Chronic intermittent hypoxia (CIH), which is characterized by cyclic hypoxic episodes of short duration followed by normoxia, is a characteristic feature of obstructive sleep apnea, the most common form of sleep disorder. The CB has been proposed to be the link between the reflex increase in sympathetic nervous system activity and the blood pressure associated with obstructive sleep apnea due to CIH (Fletcher et al., 1992; Narkiewicz et al., 1999). In fact, an augment in peripheral CB drive in obstructive sleep apnea patients has been observed, since they showed an increase in ventilatory and cardiovascular reflex responses induced by acute hypoxia (Narkiewicz et al., 1999). This increase in CB drive has been showed to be due to CIH, since Peng et al. (2003) demonstrated that CIH induced a progressive increase in CSN activity with each hypoxic episode, remaining the baseline activity elevated approximately during 60 min after the last acute hypoxic stimuli, an effect called sensory long-term facilitation. These authors also suggested that sensory long-term facilitation contributes to the persistent increase in sympathetic nervous activity and blood pressure that is observed in obstructive sleep apnea, since the increase in CB sensory activity triggers sympathetic nerve discharge and an increase in blood pressure (Peng et al., 2003). Recently, our group showed that adenosine is one of the mediators involved in the sensitization of CB during CIH (Sacramento et al., 2015), since caffeine decreased basal and hypoxia-evoked (5% O_2_) CSN chemosensory activity in rats subjected to 15 days of CIH (Sacramento et al., 2015). Moreover, it has been described that adenosine levels are augmented in obstructive sleep apnea patients (Lavie, 2003) suggesting a deregulation of adenosinergic system in sleep apnea patients. Therefore, the blockage of adenosine receptors in the CB or modulation of adenosine metabolism both in the CB and peripherally might be useful to treat some of the pathophysiological features of chronic obstructive sleep apnea.

### Hypertension

Hypertension affects one-third of the human population and in the United States only 53% of those tacking antihypertensive medication have their condition controlled (Go et al., 2014). Furthermore, it is estimated that 14–16% of all patients with hypertension are resistant to antihypertensive medication and/or having poor compliance or tolerance to the medication (Achelrod et al., 2015). It is accepted that CB chemoreflex-evoked sympathetic activity responses are increased in human patients and animal models of systemic essential hypertension (Trzebski et al., 1982; Somers et al., 1988; Tan et al., 2010; Abdala et al., 2012; Siński et al., 2012) and therefore the CB has been proposed as a therapeutic target for the treatment of cardiovascular diseases. Accordingly, CB ablation was capable of control the development and maintenance of high blood pressure in spontaneously hypertensive rats and humans (Abdala et al., 2012; Narkiewicz et al., 2016). However, the effect of unilateral ablation CB in hypertensive patients has diminished efficacy 12 months after ablation, suggesting a compensation of the remaining CB (Narkiewicz et al., 2016), suggesting that other approaches are needed to modulate CB function in cardiovascular diseases. Pijacka et al. (2016) demonstrated an upregulation of the P2X_3_ mRNA in the chemoreceptive petrosal sensory neurons of spontaneously hypertensive rats and that P2X_3_ receptors are present in human CB from individuals with a medical history of hypertension. Moreover, it has also shown that a P2X_3_ receptor antagonist is capable to decrease sympathetic activity and arterial pressure in spontaneously hypertensive rats, an effect that was absent in normotensive animals (Pijacka et al., 2016). All these data suggest that ATP is responsible for the CB hyperactivity and hyperreflexia seen in essential hypertension and that support the modulation of P2X_3_ receptor as non-surgical a non-surgical strategy to control human hypertension. Although, apart from P2X_3_ receptor, and knowing that P2X_4_ receptors are expressed post-synaptically in the CB (Campanucci et al., 2006) and that they are involved in ventrolateral medulla control of the sympathetic autonomic function (Zoccal et al., 2011), we can postulate that modulation of P2X_4_ receptors might be a therapeutic target for hypertension.

## Author Contributions

All authors listed have made a substantial, direct and intellectual contribution to the work, and approved it for publication.

## Conflict of Interest Statement

The authors declare that the research was conducted in the absence of any commercial or financial relationships that could be construed as a potential conflict of interest.
